# Advancing personalized digital therapeutics: integrating music therapy, brainwave entrainment methods, and AI-driven biofeedback

**DOI:** 10.3389/fdgth.2025.1552396

**Published:** 2025-02-25

**Authors:** Dian Jiao

**Affiliations:** Institute of Psychiatry, Psychology & Neuroscience, King’s College London, London, United Kingdom

**Keywords:** music therapy, binaural beats, multisensory stimulation, brainwave entrainment, neuroplasticity, cognitive rehabilitation, artificial intelligence, non-pharmacological interventions

## Abstract

Mental health disorders and cognitive decline are pressing global concerns, increasing the demand for non-pharmacological interventions targeting emotional dysregulation, memory deficits, and neural dysfunction. This review systematically examines three promising methodologies—music therapy, brainwave entrainment (binaural beats, isochronic tones, multisensory stimulation), and their integration into a unified therapeutic paradigm. Emerging evidence indicates that music therapy modulates affect, reduces stress, and enhances cognition by engaging limbic, prefrontal, and reward circuits. Brainwave entrainment, particularly within the gamma frequency range (30–100 Hz), facilitates neural oscillatory patterns linked to relaxation, concentration, and memory, with 40 Hz showing promise for cognitive enhancement, albeit with individual variability. Synchronized multisensory stimulation, combining auditory and visual inputs at gamma frequencies, has demonstrated potential in enhancing memory and supporting neural integrity, particularly in Alzheimer's disease. However, challenges such as patient response variability, lack of standardization, and scalability hinder widespread implementation. Recent research suggests that a synergistic application of these modalities may optimize therapeutic outcomes by leveraging complementary mechanisms. To actualize this, AI-driven biofeedback, enabling real-time physiological assessment and individualized adjustments—such as tailoring musical complexity, entrainment frequencies, and multisensory components—emerges as a promising solution. This adaptive model enhances treatment accessibility and consistency while maximizing long-term efficacy. Although in early stages, preliminary evidence highlights its transformative potential in reshaping non-pharmacological therapeutic strategies. Advancing this field requires interdisciplinary research, rigorous evaluation, and ethical data stewardship to develop innovative, patient-centered solutions for mental health and cognitive rehabilitation.

## 1 Introduction

Mental health disorders and cognitive deterioration exert considerable pressures on both individuals and healthcare infrastructures. Conventional pharmacological treatments frequently alleviate symptoms yet inadequately address the fundamental neural impairments, with adverse effects and restricted long-term effectiveness remaining ongoing obstacles (1). Consequently, researchers have increasingly investigated comprehensive, safer, and more sustainable methodologies that capitalize on the brain's plasticity, emotional regulation, and integrative sensory processing capabilities.

Music therapy is particularly noteworthy for its capacity to activate limbic, prefrontal, and sensory pathways, influencing mood and cognitive function through dopaminergic reward circuits and stress-related hormonal responses (2–5). In contrast, brainwave entrainment methods—including binaural beats, isochronic tones, and multisensory stimulation—seek to synchronize neural oscillations with frequencies correlated to desired mental states, though their effectiveness is inconsistent among individuals due to inherent biological and psychological variances (6–11). Particularly within the gamma frequency spectrum (30–100 Hz), these approaches have attracted scholarly interest for their potential to enhance memory and cognitive abilities while mitigating neurodegenerative indicators (12–14).

Notwithstanding their potential, these modalities have often been examined independently, with minimal investigation into the synergistic and enduring benefits that might arise from their integration. Obstacles to wider implementation encompass variability in patient responses, a lack of standardized protocols, and logistical impediments related to accessibility and scalability. Overcoming these challenges necessitates a paradigm shift: the incorporation of emotional-cognitive support (music therapy) and brainwave entrainment methods under adaptive AI-driven biofeedback platforms capable of dynamically customizing interventions to individual physiological, cognitive, and emotional conditions (15).

This review consolidates the prevailing evidence regarding music therapy, binaural beats, isochronic tones, and multisensory stimulation while critically assessing their underlying mechanisms, therapeutic applications, and limitations. Additionally, it proposes an integrative framework that harnesses AI-driven personalization to unify these modalities and optimize their complementary strengths. By bridging the divide between theoretical constructs, empirical investigations, and clinical practice, this approach aspires to foster patient-centered, scalable solutions for mental health treatment and cognitive rehabilitation.

## 2 Survey methodology

A systematic and structured methodology was employed to identify, select, and synthesize relevant literature, ensuring methodological rigor, thematic relevance, and inclusion of high-quality studies. This approach spans diverse disciplines, including neuroscience, psychology, rehabilitation medicine, digital health, and investigations into brainwave entrainment methods, to comprehensively examine music therapy, binaural beats, isochronic tones, multisensory stimulation, and their potential AI-driven integrative applications.

### 2.1 Literature search

The review utilized multiple electronic databases, including PubMed, Scopus, Web of Science, and Google Scholar. The searches were confined to peer-reviewed journal articles, conference proceedings, and reputable academic sources published in English. To capture foundational theories along with cutting-edge advancements, no fixed start date was imposed, although most studies have been conducted over the last two decades. Keyword combinations reflected the review's core themes: “music therapy,” “binaural beats,” “isochronic tones,” “multisensory stimulation,” “brainwave entrainment,” “gamma oscillations,” “cognitive enhancement,” “mental health,” “neuroplasticity,” and “non-pharmacological interventions,” along with terms related to “Artificial Intelligence” or “AI,” “Machine learning” or “ML”, “Artificial General Intelligence” or “AGI”, “real-time feedback,” and “adaptive therapy.”

The initial search generated several hundred sets of results. Titles and abstracts were screened systematically to exclude studies that did not report measurable cognitive, emotional, or neurological outcomes or those focusing solely on pharmacological treatments. Empirical research demonstrating quantifiable impacts on mood, anxiety, depression, attention, memory, and cognitive performance was prioritized in this study. Intervention studies, quasi-experimental designs, case series, and systematic reviews that provided mechanistic insights or clinical relevance were included. Articles that lacked methodological clarity, empirical evidence, or those addressing unrelated topics were excluded.

### 2.2 Data extraction and quality assessment

A standardized data extraction process was used to ensure transparency and reproducibility. For each study, details such as authorship, publication year, participant demographics, clinical characteristics, study design, interventions, outcome measures, and key findings were systematically recorded. Special emphasis was placed on studies examining neural mechanisms using techniques like fMRI, PET, EEG, or established biomarkers of emotional and cognitive states. This process enabled cross-study comparisons, the identification of knowledge gaps, and the recognition of patterns relevant to integrative strategies.

Quality assessment further refined the selected corpus. Preference was given to studies employing randomized controlled trials (RCTs) or robust quasi-experimental designs. Exploratory and pilot studies were included only if they offered valuable mechanistic insights or novel perspectives. The evaluation criteria included the presence of control conditions, statistical rigor, sample size adequacy, and clarity of reporting. Limitations such as small sample sizes, brief intervention durations, and reliance on subjective measures were noted to contextualize the findings critically.

### 2.3 Synthesis approach

Given the heterogeneity in study designs, populations, and intervention protocols, narrative synthesis was adopted instead of a meta-analytic structure. Evidence was integrated to highlight convergent trends, persistent challenges, and complementary strengths across music therapy, brainwave entrainment methods, and multisensory stimulation. Key themes emerged, such as the engagement of emotional circuits, the potential for frequency-based modulation of brainwave activity, enhancement of memory and attention, and scalability through digital platforms and AI adaptation. Conceptual models and emerging empirical findings proposing integrative strategies are also incorporated to bridge theoretical and practical considerations.

### 2.4 Balancing breadth and depth

This methodology balanced breadth by capturing the interdisciplinary scope of the topic and depth by rigorously scrutinizing the quality and relevance of the included studies. The synthesis situates each modality within its empirical and theoretical context, with particular attention paid to their interplay under AI-driven systems. This foundation supports the detailed analyses and forward-looking propositions explored in the subsequent sections, advancing the discussion on innovative, non-pharmacological interventions for mental health and cognitive enhancement.

## 3 Neural underpinnings of music therapy and sensory induced brainwave entrainment

### 3.1 The neuroscience of music therapy

As an emotional-cognitive support modality, music therapy encompasses a multifaceted interaction of neural circuits, utilizing the auditory, emotional, and cognitive pathways of the brain to influence mood, diminish stress, and augment cognitive performance (4, 5, 16, 17). Employing sophisticated neuroimaging modalities, such as functional magnetic resonance imaging (fMRI) and electroencephalography (EEG), scholars have demonstrated that musical experiences engage a variety of cerebral regions, including the auditory cortex for sound analysis, the amygdala and hippocampus for emotional relevance, and the prefrontal cortex for executive functioning (2, 3). In contrast to passive auditory engagement, music therapy actively modulates neural processes, fostering neuroplasticity, emotional regulation, and the encoding of memories (17, 18).

The modulation of neurochemicals constitutes a fundamental mechanism that underlies these therapeutic effects. Enjoyable or significant musical stimuli incite the release of dopamine within the mesolimbic pathway, thereby reinforcing affirmative emotional states and encouraging active participation (4, 17). Concurrently, music therapy diminishes cortisol levels, the principal hormone associated with stress, while elevating serotonin levels, which together promote relaxation and psychological resilience (2, 3). These neurochemical alterations facilitate long-term stabilization of mood, rendering music therapy particularly efficacious for individuals grappling with emotional dysregulation or mood disorders (18).

Beyond its neurochemical influences, music therapy also affects neural connectivity and oscillatory dynamics. By engaging both sensory and cognitive faculties, it reorganizes synaptic connections and fortifies networks associated with memory, attention, and executive functions (16, 19, 20). For instance, structured musical rhythms may assist stroke survivors by entraining motor networks, thereby enhancing motor recovery and improving sensorimotor integration. In geriatric populations or those with mild cognitive impairment, musical interventions activate preserved neural pathways, potentially decelerating cognitive decline and enhancing life quality (17).

A further notable advantage is the enhancement of emotional regulation. Music therapy recalibrates emotional processing by stimulating the amygdala and hippocampus, which are integral to the contextualization of emotions and memory. Individuals suffering from anxiety, depression, or post-traumatic stress may experience alleviation as music restores equilibrium between limbic reactivity and prefrontal inhibitory mechanisms (3–5). Group-based interventions, such as choral singing or drumming, amplify these therapeutic effects by synchronizing the brain rhythms of participants and fostering social connectedness and collective emotional experiences (21–23). This collective engagement not only enhances individual wellness but also bolsters social resilience.

The therapeutic capacity of music therapy aligns well with other non-pharmacological interventions. By providing a stable emotional and cognitive baseline, music therapy can complement brainwave entrainment methods (e.g., binaural beats, isochronic tones) and multisensory stimulation, potentially enhancing their efficacy (15, 20). Innovations in digital technology have further augmented this potential by facilitating real-time personalization grounded in physiological feedback. Adaptive systems have the capability to customize musical complexity, rhythm, and style to align with the specific needs of patients, thereby sustaining neuroplastic transformations while accommodating cultural and individual preferences (24, 25). Furthermore, the ability of music therapy to engage dopaminergic reward systems, modulate stress hormones, and strengthen neural connectivity provides a robust foundation for non-pharmacological mental health and cognitive interventions (2, 3, 17, 23). Its integration with AI-driven biofeedback holds promise for the development of innovative and highly individualized therapeutic frameworks (5, 16, 17).

### 3.2 Binaural beats, isochronic tones, and brainwave entrainment

Binaural beats offer a subtle yet compelling auditory intervention, where slightly different frequencies presented to each ear create the perception of an illusory “beat” absent in the actual sound waves (7, 8, 11). Similarly, isochronic tones employ a single pulsed tone that switches on and off at precise intervals, likewise intended to entrain brain activity. These methods are thought to modulate neural oscillations toward frequency bands associated with specific mental states. For instance, alpha frequencies (8–12 Hz) support relaxed alertness and stress reduction; theta ranges (4–7 Hz) are linked to meditative states and memory consolidation; and gamma frequencies (30–100 Hz) are associated with attention and higher-order cognitive processing (6, 19, 20).

Preliminary findings suggest that these brainwave entrainment techniques may influence mental states and enhance mental health and cognitive rehabilitation outcomes. In some studies, participants exposed to targeted frequencies reported reduced anxiety and improved sustained attention and working memory (6, 9). By modulating neural oscillatory activity, binaural beats and isochronic tones can complement or amplify other interventions—such as preparing the brain for relaxation before music therapy or enhancing the integration of multisensory stimulation (7, 8).

Despite these potential benefits, several significant challenges remain. The chief factor among these is the variability in individual responses. Baseline neural states, differences in auditory processing, and levels of engagement or expectancy significantly affect the effectiveness of neural entrainment (8, 10). Moreover, the lack of standardized protocols complicates generalization. Questions remain regarding the most effective frequency ranges for addressing specific conditions, optimal session durations, and whether these methods are more effective as standalone stimuli or when embedded within music or natural soundscapes (7, 11).

Methodological limitations also constrain the evidence base. Many studies have relied on small samples or pilot designs that lack control groups and robust biomarkers. For example, while EEG measures can confirm genuine shifts in alpha, theta, or gamma power, such data are rarely incorporated, leaving causal links between brainwave entrainment and cognitive-emotional benefits largely speculative (8, 26). Cultural and personal preferences add another layer of complexity. Pure tones may feel uncomfortable for some individuals, underscoring the need for personalization. Embedding beats into music or tailoring session lengths to align with user comfort can enhance adherence and effectiveness (9, 10).

By addressing these challenges through standardizing protocols, identifying target populations more clearly, and refining intervention parameters, binaural beats and isochronic tones may become powerful adjuncts to music therapy and multisensory stimulation in personalized treatment models (18, 27). Building on their conceptual framework, these auditory entrainment approaches have the potential to transition from niche interventions to widely accessible, technology-driven strategies for mental health care.

### 3.3 Multisensory stimulation and cognitive enhancement

Although music therapy and previously discussed brainwave entrainment methods (e.g., binaural beats, isochronic tones) primarily rely on auditory inputs and have been shown to modulate emotional processing (2, 3), multisensory stimulation (MSS) expands these principles by integrating auditory, visual, and sometimes tactile or proprioceptive inputs (12). Importantly, this modality capitalizes on the brain's inherent ability to synthesize multiple inputs, thereby enhancing cognitive outcomes through synchronized stimuli. For instance, rhythmic light flickers paired with corresponding auditory signals can reinforce or entrain neural oscillations in the gamma frequency range (approximately 30–100 Hz), with particular emphasis on 40 Hz, which research has linked to memory consolidation, attention regulation, and integrative perceptual processing (13, 19, 20, 28–30).

Recent empirical studies underscore the therapeutic potential of this expanded approach, demonstrating improvements in memory performance and possible mitigation of neuropathological changes observed in Alzheimer's disease (12, 13, 30, 31). Unlike pharmacological interventions, which often produce systemic side effects, MSS attempts to directly modulate brain activity in a noninvasive manner. By aligning external sensory inputs with intrinsic oscillatory dynamics, this strategy aims to reorganize neural connectivity and processing patterns toward healthier states. Beyond memory enhancement, multisensory interventions could potentially address attentional deficits, motor recovery, and emotional regulation, thereby broadening their role in neurorehabilitation (32).

Nevertheless, MSS presents notable implementation challenges. Precise temporal coordination of auditory and visual stimuli is critical because even minimal desynchronizations can weaken entrainment effects (33). Moreover, the specialized equipment and controlled environments required to deliver synchronized stimuli pose logistical and financial barriers to large-scale adoption. At the same time, individual variability adds another layer of complexity, as factors such as baseline cognitive function, sensory acuity, cultural background, and personal preferences shape outcomes (34, 35).

Despite these obstacles, MSS aligns with fundamental principles of neural integration in the brain. Humans naturally merge multiple sensory inputs to interpret their surroundings, making this modality especially resonant with day-to-day functioning. Future research could explore tailored approaches that adapt sensory combinations to specific user needs. For example, auditory and visual cues might enhance memory training for certain individuals, whereas tactile inputs can better address motor learning or emotional grounding (36, 37).

It is important to note that MSS also dovetails with music therapy and auditory entrainment methods in potentially synergistic ways. For instance, familiar music paired with 40 Hz light flickers could heighten emotional engagement, priming neural circuits for enhanced plasticity (4, 17). Likewise, embedding binaural beats in a multisensory setting may help sustain attention and achieve targeted neural states more consistently (7, 9). In essence, MSS is a holistic and innovative approach for cognitive enhancement. By emphasizing the interplay among diverse sensory pathways, it broadens the therapeutic landscape beyond purely auditory or affective channels. While early-stage research highlights technical, logistical, and individual variability challenges, the potential to combine MSS with music therapy and other brainwave entrainment methods offers an exciting frontier for neurorehabilitation, cognitive training, and emotional support (27, 38, 39).

## 4 Current challenges and limitations

Despite a growing body of evidence supporting music therapy, binaural beats, and multisensory stimulation, significant challenges limit their effectiveness, scalability, and clinical integration. One of the most prominent obstacles is marked variability in patient responses. Baseline neural states, sensory processing abilities, cultural influences, and personal preferences shape how individuals perceive and respond to these interventions (8, 34). For example, music therapy often requires careful personalization, as musical elements must resonate emotionally and cognitively with each patient, making it resource-intensive and difficult to standardize or scale (17). Similarly, binaural beats show heterogeneous effects, with some individuals experiencing relaxation or enhanced focus, whereas others report negligible changes (9, 10). Multisensory stimulation further compounds this variability, as outcomes depend on individual sensory acuity and cognitive status, along with the precise alignment of auditory, visual, and other inputs (33, 40).

Another critical limitation is the absence of standardized protocols. Few universally accepted frameworks exist to guide practitioners on matching specific musical elements, binaural frequencies, or multisensory stimuli to desired therapeutic outcomes (18). This lack of consistency hampers reproducibility complicates evidence-based practice and creates barriers for large-scale implementation. Without robust clinical guidelines, practitioners may struggle to adopt these modalities confidently, and researchers may find it challenging to compare findings across studies (15, 24).

In addition, scalability and accessibility pose substantial challenges. Delivering these therapies often requires skilled practitioners, advanced equipment, and controlled environments, which limits their reach to underserved or remote populations (39, 41). Music therapy depends on trained professionals to tailor sessions; binaural beats require high-quality audio systems and distraction-free environments; and multisensory stimulation involves specialized apparatus for precise synchronization. While digital health technologies may address some barriers, they introduce further considerations, such as user-friendliness, data privacy protection, and technical support requirements (15).

Ethical considerations regarding data governance are equally pressing. As AI-driven systems increasingly support these modalities, collecting physiological data, such as EEG signals or heart rate variability, raises concerns about informed consent, data security, and algorithmic transparency (15, 42). Notably, users must understand how their data are utilized, who has access, and whether algorithmic decisions are free from bias or hidden errors. While the prospect of dynamically adjusting interventions based on real-time neural or emotional states is promising, rigorous oversight is required to ensure fairness, accountability, and patient autonomy (41).

Moreover, theoretical gaps undermine the potential of these modalities. Although supported by empirical work—such as neural plasticity mechanisms in music therapy, frequency-specific entrainment in binaural beats, and gamma coherence in multisensory stimulation—many studies lack sufficient depth to establish causal pathways (28, 29). Research is often constrained by small sample sizes, short durations, and reliance on subjective measures, limiting conclusions about long-term safety, efficacy, or dose-response relationships (8, 26). Translational gaps further exacerbate this issue, as findings from controlled laboratory studies may not replicate real-world conditions with distractions and user fatigue.

Cost and economic barriers also complicate scalability. Developing AI-driven platforms, acquiring reliable hardware, and training clinicians or users to operate complex systems require significant investment (15). These expenses may restrict access to affluent institutions or well-resourced users, thereby perpetuating existing health disparities. Ensuring cost-effective, durable, and user-friendly solutions in diverse socioeconomic settings is critical for equitable access (18).

Finally, there is a lack of high-quality comparative research evaluating the synergy of these modalities. Few studies have systematically examined combinations such as music therapy paired with binaural beats or multisensory stimulation enhanced by AI-guided adjustments, leaving their interactive benefits largely speculative (8, 10). Hence, future interdisciplinary collaborations could address this gap by designing protocols that vary and combine modalities systematically, clarifying their neural and behavioral interactions across different conditions (15).

Addressing these challenges requires coordinated efforts across disciplines. By developing standardized guidelines, refining technological infrastructure, and investing in longitudinal studies, the field can overcome current limitations and pave the way for integrative, AI-powered therapeutic frameworks. Such advancements will enable more reliable, scalable, and equitable interventions, ultimately broadening access to nonpharmacological mental health and cognitive rehabilitation strategies (15, 18).

## 5 Future directions: integrative approaches with AI-empowered real-time feedback

Having explored the mechanisms, evidence, and limitations of music therapy, brainwave entrainment methods (including binaural beats, isochronic tones and multisensory stimulation), it is logical to propose an integrative framework that leverages machine learning–based artificial intelligence (AI) and continuous biofeedback to create adaptive, personalized interventions. While fully realized AI remains speculative, current AI algorithms can already process physiological data in real time, recognizing that mental health conditions and cognitive deficits vary widely across individuals and evolve over time (43, 44). Such machine learning–driven systems enable dynamic adjustments to therapeutic parameters, aligning with a user's changing needs and contexts.

Additionally, distinguishing machine learning (ML) from artificial general intelligence (AGI).

To begin with, ML refers to a subset of AI that focuses on algorithms trained to identify patterns in data, thereby enabling task-specific predictions or decisions without explicit reprogramming (45, 46). By contrast, AGI aims to emulate the broad cognitive flexibility of humans, encompassing complex reasoning, problem-solving, and adaptive learning across diverse domains (45). Although ML systems are already extensively applied in fields ranging from healthcare to finance, AGI remains largely theoretical and would require significant breakthroughs in our understanding of human cognition (46). Therefore, the personalized digital therapeutics proposed here rely on machine learning–based methods that can operate effectively within well-defined parameters. Ultimately, the possibility of AGI might broaden these real-time adaptive therapies, but it is not yet feasible for immediate clinical application. In light of these constraints, the present framework leverages machine learning capabilities that are already attainable, particularly in the realm of real-time monitoring and adaptive feedback.

Furthermore, at the heart of this vision lies AI-empowered real-time monitoring. Continuous data streams collected from wearable sensors, EEG recordings, heart rate variability monitors, or self-report apps provide a detailed, moment-to-moment understanding of an individual's physiological and emotional state (47, 48). These data inform AI algorithms to detect patterns—such as elevated stress markers, lapses in attention, or mood shifts—and adapt interventions accordingly. For example, if binaural beats fail to achieve alpha-wave relaxation, the system can modify the frequency range or transition into a soothing musical sequence. Similarly, if memory outcomes during multisensory stimulation remain suboptimal, adjusting gamma-frequency alignment or stimulus intensity may enhance neural entrainment (49, 50).

By integrating multiple modalities, AI-driven systems can unlock new opportunities for synergy. The ability of music therapy to foster emotional engagement primes neural circuits for enhanced attentional states, potentially amplified by binaural beats or isochronic tones. Meanwhile, multisensory stimulation reinforces neural oscillations such as gamma rhythms, thereby consolidating cognitive gains (43, 44). As summarized in Table 1, these approaches differ in mechanisms, required engagement, and adaptability, but AI-driven biofeedback system can dynamically tailor each to individual needs, thus overcoming the limitations of static, one-size-fits-all interventions.

**Table 1:** Comparative overview of music therapy, brainwave entrainment methods, and AI-driven biofeedback systems.

Modality	Neural activation	Primary mechanism	User engagement	Therapeutic potential	Key challenges
Music therapy (MT)	Primary: Limbic system (emotional regulation), prefrontal cortex (cognitive control), and reward circuits (pleasure and motivation) Secondary: Auditory cortex (sound processing)	Engages emotional circuits through culturally relevant music that evokes memories and emotional responses. This activates the dopaminergic reward pathways, promoting feelings of pleasure and motivation. It also modulates stress hormones like cortisol, aiding in relaxation and emotional regulation.	High; music is emotionally resonant, culturally adaptable	- Mood regulation - Stress reduction - Cognitive enhancement - Social support (Broad impact through emotional engagement)	- Requires trained facilitators - Resource-intensive - Standardization challenges - Individual preference variation
Binaural beats (BB)	Primary: Auditory pathways (brainstem, including the inferior colliculus and superior olivary complex) Secondary: Potential involvement of cortical areas (e.g., prefrontal cortex for attention) and frequency-following response pathways	Creates the perception of a third tone through inter-aural frequency differences, occurring when slightly different frequencies are presented to each ear. This phenomenon is theorized to entrain specific brainwave patterns (e.g., alpha for relaxation, beta for focus, gamma for heightened cognition). However, empirical evidence is mixed, with some studies supporting brainwave entrainment and others finding no significant effects.	Moderate; can be embedded in music to enhance engagement	- Focused attention - Relaxation - Memory enhancement (Specific outcomes through frequency targeting)	- Individual response variability - Limited standardization - Requires controlled listening conditions - Need for more robust evidence
Isochronic tones (IT)	Primary: Auditory cortex (processing of rhythmic tones) Secondary: Limited evidence suggests possible effects on attention networks.	Involves a single pulsed tone delivered at specific intervals, producing sharp neural response patterns in the auditory cortex. Unlike BB, IT does not require headphones, making it easier to deliver. The rhythmic pulses are believed to synchronize brainwave activity, potentially enhancing attention, relaxation, or memory consolidation. However, research on IT is limited, and more studies are needed to confirm these effects.	Moderate; more noticeable than BB, may affect compliance	- Evidence suggested more effective for some individuals (Provides alternative to BB)	- Fewer studies than BB - User comfort issues - Limited long-term data - Standardization needed
Multisensory stimulation (MSS)	Primary: Multisensory integration areas (e.g., superior colliculus, visual cortex, auditory cortex, and somatosensory cortex). Secondary: Hippocampus (memory-related processing) and prefrontal integration areas (cognitive control, decision-making, and attention)	Combines synchronized sensory inputs, such as light, sound, and touch, to target specific neural oscillations (e.g., gamma ∼40 Hz). Research suggests that 40 Hz MSS can enhance cognitive functions by promoting neural entrainment and stimulating regions like the prefrontal cortex and hippocampus. It is often explored for its potential in Alzheimer's treatment and cognitive enhancement.	Moderate to High; immersive but risk of sensory fatigue	- Enhanced memory - Motor rehabilitation - Cognitive integration (Leverages natural sensory integration)	- Equipment costs - Complex setup - Potential sensory overload - Individual tolerance varies
AI-driven biofeedback systems	Multi-regional targeting with real-time neural adaptation. These systems activate specific regions such as the prefrontal cortex (for cognitive control and decision-making), anterior cingulate cortex (for attention and error monitoring), and limbic structures (for emotional regulation and stress response).	Real-time physiological monitoring (EEG, HRV) with adaptive stimulus adjustment	Very High; interactive, personalized, mobile/wearable options	- Enhanced effectiveness of other modalities - Personalized intervention - Consistent optimization (Maximizes therapeutic impact)	- Data privacy concerns - Technical complexity - Cost barriers - Algorithm bias risks

The adaptability of AI-enhanced systems is especially effective in overcoming current challenges. By leveraging real-time feedback, these systems address variability in user responses, ensuring greater consistency across sessions. Additionally, AI integration with mobile and wearable devices significantly boosts accessibility and scalability. This innovation allows interventions to extend beyond clinical environments, diminishing reliance on trained professionals and controlled settings, thus making personalized therapies more widely available (43, 51, 52).

**Figure 1:**
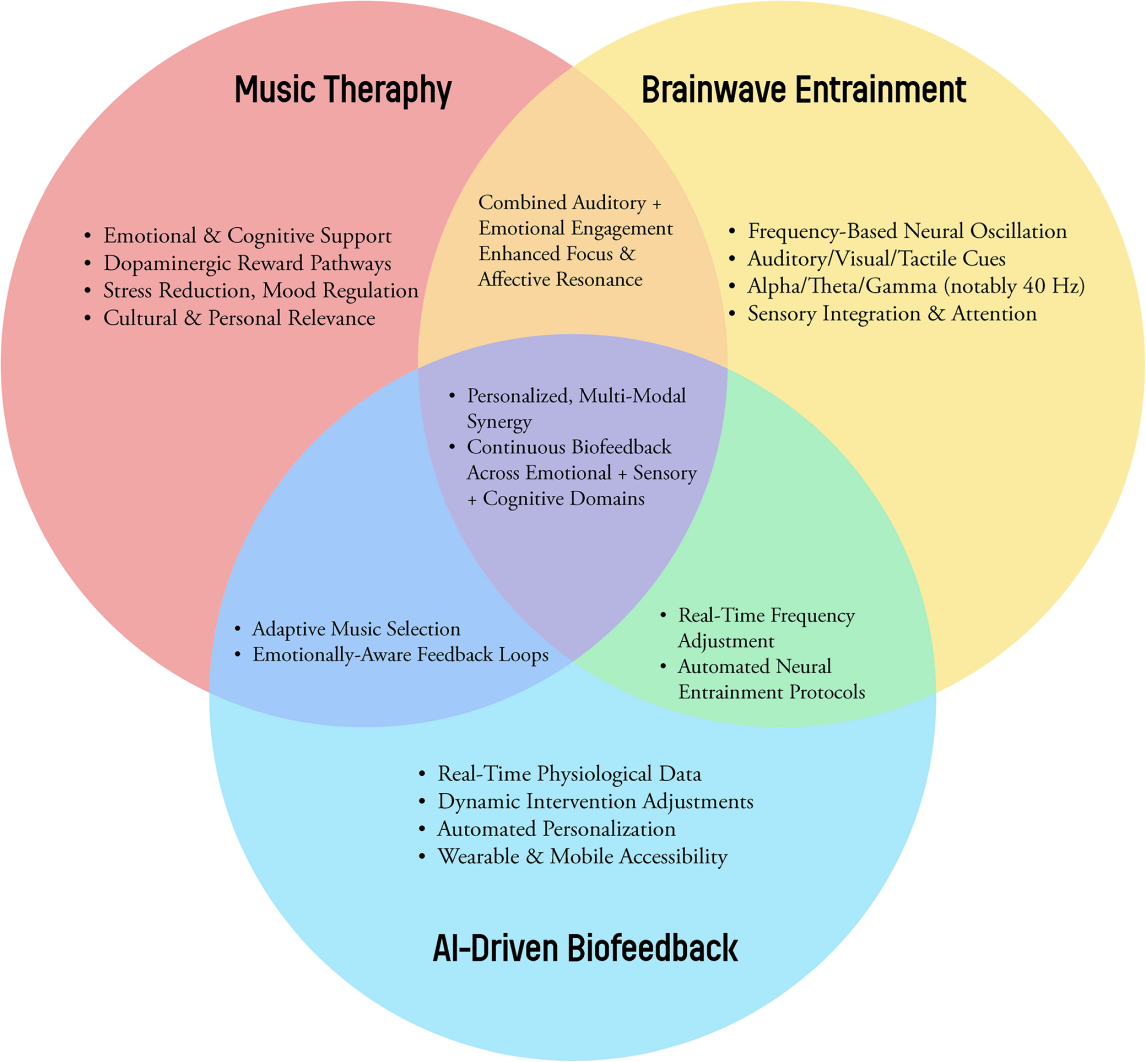
A conceptual Venn diagram illustrating the synergy among music therapy, brainwave entrainment methods (binaural beats, isochronic tones, multisensory stimulation), and AI-driven biofeedback.

Furthermore, AI integration facilitates large-scale data collection of therapeutic protocols (Figure 1). As anonymized datasets expand, machine learning models can discern which combinations of interventions work best for specific populations under various conditions (47, 53). This iterative refinement process—deploying interventions, analyzing outcomes, and updating algorithms—can establish evidence-based guidelines, enhance reproducibility, and create standardized therapeutic frameworks (44, 48).

Nevertheless, ethical considerations must remain paramount. Individuals must consent to data collection and comprehend its usage, while developers ensure secure handling of sensitive physiological and emotional data (52, 54). Bias in AI systems poses additional risks, as underrepresentation in training datasets can yield unequal benefits across demographic groups (42). Transparency in algorithm design, routine audits, and stakeholder engagement are vital for equitable implementation. Regulatory bodies must establish guidelines that balance innovation with safety, autonomy, and fairness (44).

Technical challenges also warrant further attention. Developing robust biofeedback loops that respond promptly to changing signals requires precise engineering to prevent overstimulation or user discomfort. For instance, abruptly shifting modalities may confuse users, highlighting the need for seamless transitions and gradual adjustments (55). The reliability and stability of hardware and software are equally crucial, as interruptions can undermine trust and engagement. Moreover, user-centered design ensures that platforms remain intuitive and accessible to diverse populations (52, 56).

A further benefit is the interdisciplinary scope of AI-driven integrative therapies. Neuroscientists can identify neural signatures that predict optimal responses to specific interventions, while psychologists contribute insights into motivational strategies that enhance user engagement (48, 57). Engineers and data scientists refine algorithms, clinicians align protocols with established practices, and ethicists guide responsible development (58). This collaborative approach enables cohesive advancement across research and practice (59, 60).

Initial efforts toward these integrative models have already demonstrated feasibility. For example, pilot studies have used EEG signals to adjust cognitive training tasks or integrated heart rate variability feedback into relaxation exercises (61). In the next phase, scaling these initiatives—combining them with music therapy, binaural beats, isochronic tones, and multisensory stimulation—and testing them in rigorous clinical trials are logical steps forward. Multisite, long-term studies involving heterogeneous populations and robust control conditions are especially important for validating efficacy, user satisfaction, and real-world functionality (53, 62).

Ultimately, the future of non-pharmacological mental health and cognitive interventions lies in dynamic, AI-driven systems. By merging music therapy's emotional richness, the oscillatory precision of binaural beats and isochronic tones, and the integrative scope of multisensory stimulation, personalized, adaptive solutions can be crafted (43, 44, 50). Although these concepts remain aspirational, their foundations are rapidly taking shape, offering a transformative shift in how mental health and cognitive challenges are addressed, optimized, and overcome.

## 6 Conclusions

The integration of music therapy, brainwave entrainment methods such as binaural beats, isochronic tones, and multisensory engagement, enhanced by adaptive artificial intelligence technologies, holds transformative potential for non-pharmacological approaches to mental health and cognitive rehabilitation. Each modality offers distinct advantages: music therapy engages emotional and cognitive pathways to alleviate mood disorders and build resilience; auditory entrainment guides neural oscillations toward frequencies associated with relaxation, focus, and memory retention; and multisensory stimulation harmonizes multiple inputs, fostering gamma oscillations linked to higher-order cognitive functions. Within a unified therapeutic framework, these modalities can create synergistic effects that surpass the efficacy of isolated interventions.

However, achieving this vision requires overcoming persistent barriers. Notably, variability in patient responses, the lack of standardized protocols, and the logistical challenges of scaling these interventions to diverse populations and real-world contexts limit their current applicability. Targeted interdisciplinary research is essential to address these limitations. Efforts must focus on refining methodologies, developing validated guidelines, and conducting rigorous longitudinal studies to establish a robust evidence base. The incorporation of ethically designed AI-driven biofeedback systems offers a promising solution. Such systems can adapt interventions dynamically, leveraging real-time physiological feedback, cultural considerations, individual preferences, and evolving clinical needs. This adaptability enhances therapeutic efficacy and patient satisfaction while also improving accessibility, particularly as mobile and wearable technologies reduce infrastructural barriers.

The successful implementation of these integrative, AI-enabled therapies depends on establishing strong ethical and regulatory frameworks. Transparent data governance, equitable algorithmic design, and safeguards for patient privacy, autonomy, and safety are critical to ensure these innovations align with the highest standards of care. Collaborative efforts among neuroscientists, clinicians, psychologists, engineers, music therapists, ethicists, and policymakers will be essential to anchor these advances in clinical realities while addressing economic and infrastructural challenges. By prioritizing equity, these solutions can democratize access to effective mental health and cognitive rehabilitation interventions, rather than exacerbating existing health disparities.

The next phase of research must focus on systematically evaluating combined interventions. Key priorities include identifying optimal modality combinations, determining patient characteristics that predict responsiveness, and validating these approaches through large-scale, multi-site clinical trials. Moving beyond proof-of-concept studies, community-based implementations can help establish the real-world feasibility and sustainability of these interventions. By consolidating disparate yet promising therapies into an evidence-based and scalable paradigm, their therapeutic potential can be maximized.

Ultimately, embracing the synergy between music therapy, binaural beats, isochronic tones, and multisensory stimulation—augmented by AI-driven personalization—represents a paradigm shift in non-pharmacological treatments. This patient-centered framework not only has the potential to enhance mental health and cognitive resilience but also stands to revolutionize neurorehabilitation and preventive healthcare. By refining theoretical models, building comprehensive datasets, and advancing adaptive technologies, the field moves closer to a future where interventions are scientifically rigorous, ethically managed, and tailored to the complexities of individual needs. Parallel to these advancements, emerging initiatives in biofeedback art—where real-time physiological signals drive artistic or musical outputs—further underscore the creative potential of integrating binaural beats, music therapy, and visual elements. Beyond conventional clinical applications, such approaches can foster deeper engagement, emotional resonance, and heightened self-awareness. The incorporation of biofeedback art into future research protocols holds promise not only for enhancing treatment adherence but also for enabling the exploration of novel, immersive therapeutic paradigms that capitalize on the synergy between multisensory stimulation and artistic expression. In this way, the evolving landscape of neurotherapeutic interventions seeks to honor the diversity of human experiences while advancing the universal goal of enhancing well-being.
